# A234 DOES FECAL CALPROTECTIN CORRELATE WITH INFLAMMATION ON ULTRASOUND IN CROHN’S DISEASE STRICTURES?

**DOI:** 10.1093/jcag/gwad061.234

**Published:** 2024-02-14

**Authors:** V Gulhati, R E Rosentreter, M Motamedi, A Macci, R Ingram, G G Kaplan, C Ma, C Seow, K Novak, R Panaccione, C Lu

**Affiliations:** University of Calgary Cumming School of Medicine, Calgary, AB, Canada; University of Calgary Cumming School of Medicine, Calgary, AB, Canada; University of Calgary Cumming School of Medicine, Calgary, AB, Canada; University of Calgary Cumming School of Medicine, Calgary, AB, Canada; University of Calgary Cumming School of Medicine, Calgary, AB, Canada; University of Calgary Cumming School of Medicine, Calgary, AB, Canada; University of Calgary Cumming School of Medicine, Calgary, AB, Canada; University of Calgary Cumming School of Medicine, Calgary, AB, Canada; University of Calgary Cumming School of Medicine, Calgary, AB, Canada; University of Calgary Cumming School of Medicine, Calgary, AB, Canada; University of Calgary Cumming School of Medicine, Calgary, AB, Canada

## Abstract

**Background:**

Fibrostenotic Crohn’s Disease (CD) is a challenging phenotype particularly due to the absence of intestinal anti-fibrotic therapies. Differentiating between strictures that are predominantly fibrotic as opposed to inflammatory remains a diagnostic dilemma. The ability to make this differentiation is critical to inform decisions for therapeutic approach. Fecal calprotectin (FC) is a stool marker reflective of intestinal inflammation. Very few studies have evaluated the relationship of FC concentration in ileal CD strictures and parameters of inflammation on intestinal ultrasound (IUS). Strictures on imaging are defined as 1) increased bowel wall thickness (BWT), 2) narrowed luminal apposition, and 3) pre-stenotic dilation (PSD). BWT and hyperemia (color Doppler signal (CDS)) are the most sensitive markers for CD inflammation on IUS. It is predicted that FC will match CDS in ileal strictures, similar to non-stricture phenotypes.

**Aims:**

We aim to correlate FC levels with IUS inflammation of ileal CD strictures.

**Methods:**

We performed a retrospective cohort pilot study exploring the relationship between FC levels and IUS inflammatory parameters in ileal strictures. FC levels were obtained ≤ 60 days of index IUS in fibrostenotic ileal CD patients. Individuals who underwent medication changes or experienced a clinical flare during this period were excluded. Inflammation was measured as BWT and CDS using a modified Limberg (ML) score. Pearson correlation for continuous variables, Spearman rank correlation and a Kruskal-Wallis test for FC and Limberg scale were completed.

**Results:**

A total of 25 fecal samples were obtained from 17 patients with ileal strictures (47% male, median age 59 years (range 18-76)) were assessed. Median FC concentrations was 204.9 ug/g, IQR: 250.4. Median ileal stricture BWT was 7.0 mm (range 3.0–10.0). 40% (10/25) had ML1 (short chains in bowel), 32% (8/25) ML2 (long chains in bowel), and 28% (7/25) ML3 (long chains and perienteric fat). There was no correlation between FC and BWT (r= .02, p = 0.92), nor FC with ML scores (r=0.20, p= 0.25). In those with ML1, median FC was 232.4, while those with ML2 or 3, had a FC of 155.6 and 469.7, respectively. FC values were significantly different between the ML scores, pampersand:003C0.0001.

**Conclusions:**

FC levels were not correlated with inflammatory parameters as seen on IUS in ileal CD. This unexpected finding may be due to ML2 scores having lower FC than anticipated, and small sample size. Other imaging factors such as loss of wall stratification need to be taken into account, and are perhaps more reflective of inflammation than BWT and CDS. This study provides the initial data to assess accuracy of FC and hyperemia of ileal CD strictures on IUS compared to histologic measures of inflammation on resected specimens.

**Funding Agencies:**

None

